# Effect of Intermittent Cold Exposure on Brown Fat Activation, Obesity, and Energy Homeostasis in Mice

**DOI:** 10.1371/journal.pone.0085876

**Published:** 2014-01-17

**Authors:** Yann Ravussin, Cuiying Xiao, Oksana Gavrilova, Marc L. Reitman

**Affiliations:** 1 Diabetes, Endocrinology, and Obesity Branch, National Institute of Diabetes and Digestive and Kidney Diseases, National Institutes of Health, Bethesda, Maryland, United States of America; 2 Mouse Metabolism Core, National Institute of Diabetes and Digestive and Kidney Diseases, National Institutes of Health, Bethesda, Maryland, United States of America; State University of Rio de Janeiro, Biomedical Center, Institute of Biology, Brazil

## Abstract

Homeotherms have specific mechanisms to maintain a constant core body temperature despite changes in thermal environment, food supply, and metabolic demand. Brown adipose tissue, the principal thermogenic organ, quickly and efficiently increases heat production by dissipating the mitochondrial proton motive force. It has been suggested that activation of brown fat, via either environmental (i.e. cold exposure) or pharmacologic means, could be used to increase metabolic rate and thus reduce body weight. Here we assess the effects of intermittent cold exposure (4°C for one to eight hours three times a week) on C57BL/6J mice fed a high fat diet. Cold exposure increased metabolic rate approximately two-fold during the challenge and activated brown fat. In response, food intake increased to compensate fully for the increased energy expenditure; thus, the mice showed no reduction in body weight or adiposity. Despite the unchanged adiposity, the cold-treated mice showed transient improvements in glucose homeostasis. Administration of the cannabinoid receptor-1 inverse agonist AM251 caused weight loss and improvements in glucose homeostasis, but showed no further improvements when combined with cold exposure. These data suggest that intermittent cold exposure causes transient, meaningful improvements in glucose homeostasis, but without synergy when combined with AM251. Since energy expenditure is significantly increased during cold exposure, a drug that dissociates food intake from metabolic demand during cold exposure may achieve weight loss and further metabolic improvements.

## Introduction

Cold exposure in mammals elicits behavioral and physiological responses that minimize heat dissipation (e.g. vasoconstriction, huddling) and increase heat generation (e.g. shivering, activation of brown adipose tissue [BAT]) [1]. The demonstration that adult humans have functional and inducible brown adipocytes [2]–[4] caused a resurgence of interest in BAT regulation and the effects of cold exposure on metabolism and metabolic regulation. In addition, inducible forms of BAT (termed beige or brite adipose tissue) arising within white adipose tissue and with distinct developmental lineages were recently identified [5]. These discoveries have led to the notion that simply increasing BAT quantity and/or function may reduce body weight by dissipating excess calories [6]–[8]. Transgenic [9], BAT transplantation [10], and β3-adrenergic agonist [11] murine studies support this claim, yet these experiments all cause supra-physiologically elevated levels of uncoupling protein 1 (Ucp1) and/or BAT mass. β3-adrenergic agonist stimulation in dogs, mice and rats can cause significant reductions in adiposity [12]. Yet all of the above approaches are non-physiological manipulations that bypass counter-regulatory adaptations that are likely activated during normal cold-induced UCP1 up-regulation [9]. Continuous cold exposure experiments in rodents have yielded conflicting results, with some studies showing decreased weight gain in rats exposed to cold [13], [14] and others showing no alteration in body weight [15].

Inspired by the beneficial effects of exercise, an intrinsically intermittent process, we explore the effects of intermittent cold exposure. Intermittent cold exposure experiments have been conducted in rodents but many of these experiments assessed the changes in cold tolerance, heat loss parameters [16], [17], and feeding behavior, not specifically whether cold ameliorates the negative health consequences of the obese state. Whether increases in the endogenous activation of BAT via environmental stimuli (e.g. cold exposure) can cause metabolic improvements in a diet-induced obese (DIO) murine model is not known.

Modulating ambient temperature in order to affect metabolism is an attractive idea that has recently gained traction. It has been suggested that cooler temperatures in human living spaces could reduce body weight and ameliorate comorbidities of obesity [6] by increasing the amount and/or activity of BAT. The goal of the present study was to assess the metabolic effects of intermittent cold exposure in DIO C57BL/6J mice.

## Materials and Methods

### Animals and diet

Fourteen-week-old DIO C57BL/6J male mice that had been fed a high-fat diet starting at 6 weeks of age (D12492, 60% kcal fat, 5.24 metabolizeable kcal/g; Research Diets, New Brunswick, NJ) were purchased from Jackson Laboratory (Bar Harbor, ME). At NIH, animals were individually housed at 22–24°C with a 12∶12-h dark-light cycle (lights on at 0600h) in a clean, conventional facility in plastic pens with wood-chip bedding and *ad-libitum* access to D12492 diet and water. The protocol was approved by the NIDDK Institutional Animal Care and Use Committee.

### Study design

#### ICE#1 and ICE#2

Following a 4-week acclimatization period, the mice were ranked by weight and distributed into three groups (8 mice/group) of equal mean body weight. On days of cold exposure (Monday, Wednesday, Friday), body weight and food intake were measured and cages were transferred to a 4°C room for the indicated number of hours (intermittent cold exposure: ICE). Control mice were similarly moved to a new room at 22°C for 1 hour (ICE#1) or 4 hours (ICE#2). Body composition (fat mass and fat-free mass) was measured by time domain Echo MRI 3-in-1 (Echo Medical Systems, Houston, TX) every 2 weeks in early morning.

#### ICE#3

Following a 4-week acclimatization period, the mice were ranked by weight and distributed into four groups (6 mice/group) of equal mean body weight. Two groups were exposed to 4°C for 4 hours three times per week while 2 control groups were treated similarly at 22°C (control: CON). Mice were treated daily with either vehicle or AM251 (3 mg/kg in 5% DMSO/5% Tween 80 in saline; Cayman Chemical, Ann Arbor, MI) by oral gavage. Body weight and food intake were measured daily prior to gavage.

At the end of the studies, mice were administered ketamine (100 mg/kg) and xylazine (10 mg/kg) by ip injection, blood was obtained by retro-orbital bleed, and tissues were removed, weighed, and frozen at −80°C until assayed.

### Total energy expenditure

Average total energy expenditure (TEE) was calculated using the energy balance technique (caloric intake minus change in body energy stores) [18]. In brief, the body composition caloric equivalents (fat mass, 9.4 kcal/g; fat-free mass, 1.0 kcal/g) are used to correct for body composition changes. The increase in body kcal content is subtracted from the total metabolizeable energy intake, yielding the TEE, which is divided by the experiment duration (ICE#1 – 77 days; ICE#2 – 74 days; ICE#3 – 28 days) to give the average daily TEE.

### CL316243 treatment

CL316243, a selective β3-adrenoceptor agonist was used to maximally stimulate facultative thermogenesis during indirect calorimetry [19]. These experiments were performed at thermoneutrality (30°C) to eliminate endogenous BAT activation. Mice were placed in a 12-chamber Environment Controlled CLAMS (Columbus Instruments, Columbus, OH) early in the morning (0645h), acclimatized for ∼4 hours, treated with CL316243 (100 µg/kg in saline, ip), and energy expenditure was measured for 5 more hours. Mice had *ad libitum* access to food and water during the entire testing period.

### Intra-peritoneal glucose tolerance test (ipGTT)

Mice were fasted overnight, glucose (1 g/kg body weight, ip) injected at 0900h, and tail blood glucose measured (Glucometer Contour, Bayer, Mishawaka, IN) at 0, 15, 30, 60, and 120 minutes post-injection. The ipGTT was conducted one day following cold exposure in ICE#1 and ICE#3 and two days after cold exposure in ICE#2 to probe the duration of cold exposure's effects. In ICE#2, insulin concentrations were also determined at 0, 15, and 120 minutes by RIA (Millipore, St. Charles, MO).

### Insulin tolerance test (ITT)

Non-fasted mice were injected with 0.75 U/kg body weight insulin (Humulin, Eli Lilly, Indianapolis, IN) at 0900h. Tail blood glucose concentrations were measured at 0, 15, 30, 45, 60, and 120 minutes post-injection.

### Glucose uptake

In ICE#1, *in vivo* glucose uptake was measured by ip injection of [1-^14^C]2-deoxyglucose (10 µCi, Perkin Elmer, Boston MA) one hour prior to termination of mice. Mice were injected at initiation of cold exposure in the 1 h group and 3 hours after the start of the cold exposure in the 4 h group. Sixty minutes post injection, two muscles (quadriceps and gastrocnemius), two fat pads (inguinal and epididymal), interscapular BAT, and the spleen were removed, weighed, homogenized, and the [^14^C]2-deoxyglucose 6-phosphate was extracted using Poly-Prep Prefilled Chromatography Columns (Bio-Rad Laboratories, Cat: 731-6211, Hercules, CA) and quantitated using Beckman Liquid Scintillation Counter [20].

### Serum hormone and metabolite profiles

Blood from retro-orbital bleeding was put in BD Microtainer Serum Separator Tubes (Becton, Dickinson and Company, Franklin Lakes, NJ), allowed to clot for 10 minutes at room temperature, spun at 10,000 rpm for 6 minutes, and serum was frozen until assayed. Free fatty acids (Roche Diagnostics Gmbh, Mannheim, Germany), triglycerides (Pointe Scientific Inc., Canton, MI), cholesterol (Thermo Scientific, Middletown, VA), β-hydroxybutyrate (BioVision, Milpitas, CA), and D-lactate (BioVision, Milpitas, CA) were measured by colorimetric assays. Serum glucose was measured by Glucometer Contour. Insulin (Millipore, St. Charles, MO), adiponectin (Millipore, St. Charles, MO), insulin-like growth factor 1 (ALPCO, Salem, NH), T3 (DiaSorin Inc, Stillwater, MN), and T4 (DiaSorin Inc, Stillwater, MN) were measured by RIA. Leptin (R&D Systems, Minneapolis, MN) and fibroblast growth factor 21 (Millipore, St. Charles, MO) were measured by ELISA. All assays were conducted as per manufacturer's protocol.

### Gene expression analyses

RNA was extracted (Qiagen RNeasy Plus Mini Kit, Germantown, MD), converted to cDNA (Roche Transcriptor High Fidelity cDNA Synthesis Kit, Indianapolis, IN), and quantitated by real-time polymerase chain reaction (qRT-PCR, Applied Biosystems 7900HT, Foster City, CA). Both SYBR green-based and taqman primer probe systems were used.

### Statistical analyses

Data are expressed as group mean ±SE. Statistical analyses were performed using JMP (version 9; SAS, North Carolina). For ICE#1 and ICE#2, one-way ANOVAs were conducted using mouse group as independent variables (CON, 1 h, 4 h for ICE#1 and CON, 4 h, 8 h for ICE#2). For ICE#3, two-way ANOVAs used cold exposure (ICE or CON) and drug treatment (AM or VEH) as grouping variables. For significant ANOVA results, a Tukey HSD post-hoc test was conducted to compare among groups. Statistical significance was prospectively defined as P<0.05.

## Results

### Cold exposure increases energy expenditure and food intake, without altering body weight

**Figure 1:**
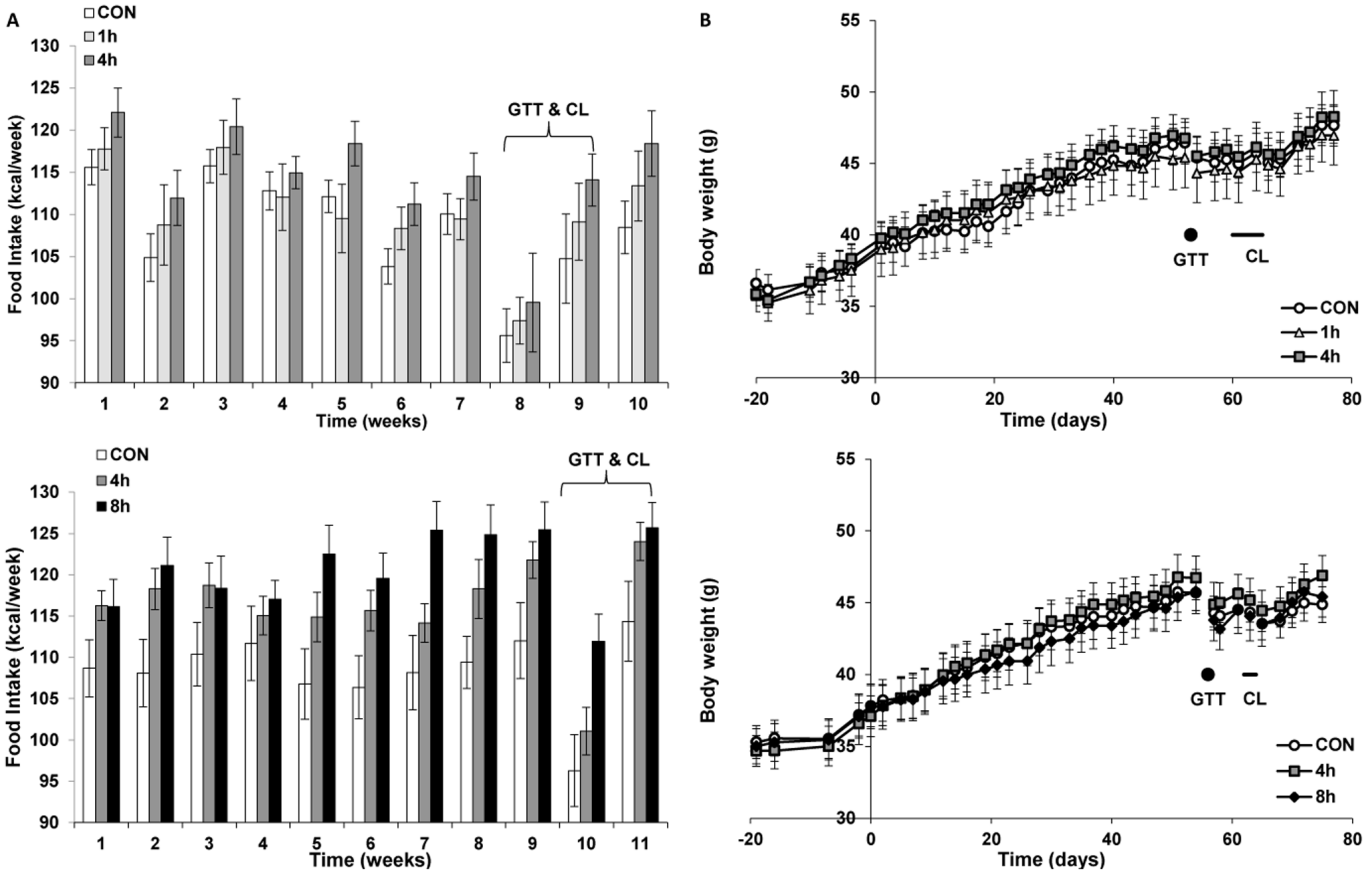
Cold exposure increases food intake, but not body weight. A: Caloric intake and B: body weight during ICE#1 (top) and ICE#2 (bottom) experiments were measured three times per week on days of cold exposure. Timing of the intra-peritoneal glucose tolerance tests (GTT) and CL316243 treatment (CL) are indicated. Data are mean ±SE, N = 8/group. The 8 h group had higher food intake as assessed by repeated measures ANOVA (p<0.05).

Two independent experiments, exposing mice to 4°C for 0, 1, or 4 hours (ICE#1) or 0, 4, or 8 hours (ICE#2) three times a week for ∼10 weeks, are presented together but were performed sequentially. This design tests a range of cold exposure durations and allows sufficient time so that metabolic and physiologic changes in the mice are likely in a new steady state. Food intake was increased in the cold exposed groups in a dose-dependent manner (**Figure 1A**). However, body weight was not affected by intermittent cold exposure (**Figure 1B**). Similarly, no consistent differences were observed in fat mass, fat-free mass, inguinal WAT, epididymal WAT, interscapular BAT, or liver weights (**Tables 1** **,** **2**). TEE over the ∼10 weeks was calculated from the caloric intake corrected for changes in body composition [18]. Cold exposure of 1 and 4 hours increased TEE by 4.5% and 7.2%, respectively (ICE#1; **Table 1**) and 4 and 8 hours increased it by 7.6% and 12.2% (ICE#2; **Table 2**). Assuming that all of the metabolic rate increase occurs during cold exposure, these data indicate an approximate doubling of metabolic rate during the cold challenge. Taken together, these data demonstrate that modest doses of intermittent cold exposure do not alter body weight or adiposity since increases in food intake fully compensate for the cold-induced increases in energy expenditure.

**Table 1:** ICE#1, effect of 1 hour and 4 hours of cold exposure three times per week.

	CON	1 h	4 h
**Body Weight (g)**	47.7±1.5	46.9±2	48.3±1.8
**Fat Mass (g)**	18.7±1.1	18.9±1.4	19.9±1.1
**Fat Free Mass (g)**	28.4±0.6	27.5±0.9	28.2±0.8
**Inguinal WAT mass (g)**	2.94±0.21	2.94±0.28	3.27±0.16
**Epididymal WAT mass (g)**	2.61±0.13	2.28±0.15	2.29±0.06
**Interscapular BAT mass (g)** *	1.09±0.04	1.10±0.07	1.17±0.06
**Liver mass (g)**	2.50±0.15	2.69±0.24	2.53±0.17
**TEE (kcal/24 h)**	14.77±0.23^A^	15.43±0.22^AB^	15.83±0.24^B^
**BAT Pgc1α RNA**	1.00±0.08^A^	1.58±0.28^A^	4.06±0.86^B^
**BAT Ucp1 RNA**	1.00±0.04^A^	1.25±0.15^AB^	1.25±0.14^B^
**WAT Pgc1α RNA**	1.00±0.16	0.67±0.12	0.79±0.09
**WAT Ucp1 RNA**	1.00±0.22	0.82±0.31	1.03±0.36
**Free fatty acids (mM)**	0.16±0.01^A^	0.23±0.03^B^	0.24±0.03^B^
**Triglyceride (mg/dl)**	41.8±3.1	52.3±4.6	45.5±6.9
**Serum Glucose (mg/dl)**	328±21	331±17	352±14
**Cholesterol (mg/dl)**	166±12	176±12	179±8
**Insulin (ng/ml)**	0.94±0.21	0.84±0.26	0.73±0.09
**Leptin (ng/ml)**	111±7^A^	101±11^AB^	89±6^B^

Tissue weights, mRNA levels, and serum analytes were taken at the end of the experiment. TEE was calculated as described in Materials and Methods using the energy balance method over the full 77 days of the experiment. Gene expression values are reported relative to CON. Data are mean ±SE, N = 8/group. Levels not connected by same letter are significantly different (P<0.05).

includes adherent WAT.

**Table 2:** ICE#2, effect of 4 hours and 8 hours of cold exposure three times per week.

	CON	4 h	8 h
**Body weight (g)**	44.9±1.2	46.9±1.4	45.4±1.4
**Fat Mass (g)**	14.4±0.76	16.33±1.23	15.54±0.8
**Fat Free Mass (g)**	25.36±0.84	25.03±0.61	25.14±0.81
**Inguinal WAT mass(g)**	2.52±0.16	2.65±0.26	2.65±0.11
**Epididymal WAT mass (g)**	1.71±0.23^A^	2.45±0.17^B^	2.48±0.07^B^
**Interscapular BAT mass(g)**	0.35±0.03	0.47±0.05	0.40±0.05
**Liver mass (g)**	1.86±0.13	1.65±0.13	1.51±0.12
**TEE (kcal/24 h)**	15.21±0.20^A^	16.37±0.21^B^	17.07±0.20^C^
**BAT Pgc1α RNA**	1.00±0.39^A^	4.64±1.32^B^	7.27±1.32^C^
**BAT Ucp1 RNA**	1.00±0.22	1.23±0.12	1.26±1.26
**WAT Pgc1α RNA**	1.00±0.24	0.60±0.27	2.72±0.94
**WAT Ucp1 RNA**	1.00±0.67	5.12±4.50	10.40±5.17
**Free fatty acids (mM)**	0.33±0.04^A^	0.61±0.11^B^	0.91±0.14^C^
**Triglycerides (mg/dl)**	59.2±7.5	50.6±9.5	69.9±10.4
**Serum Glucose (mg/dl)**	315±24	332±27	275±16
**Cholesterol (mg/dl)**	149±7	156±10	149±5
**Insulin (ng/ml)**	1.39±0.13^A^	1.19±0.09^AB^	1.07±0.05^B^
**Adiponectin (µg/ml)**	15.5±1.2	16.5±0.8	15.7±1.0
**Leptin (ng/ml)**	86±10	75±8	74±4

Tissue weights, mRNA levels, and serum analytes were taken at the end of the experiment. TEE was calculated as described in Materials and Methods using the energy balance method over the full 74 days of the experiment. Gene expression values are reported relative to CON. Data are mean ±SE, N = 8/group. Levels not connected by same letter are significantly different (P<0.05).

### BAT activation by cold exposure

**Figure 2:**
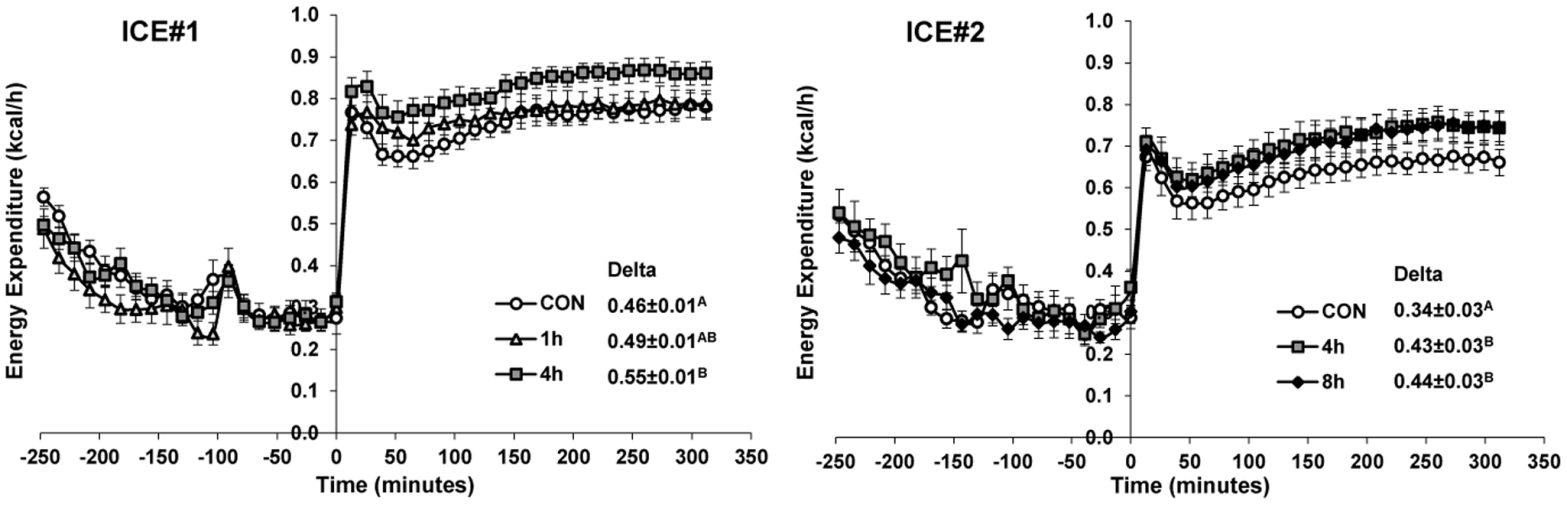
Prior cold exposure increases CL316243-induced energy expenditure. In ICE#1 (top) and ICE#2 (bottom), mice were placed in the individual chambers at 30°C at −250 minutes, injected CL316243 (100 µg/kg) at time 0 as described in Experimental Procedures. Energy expenditure was measured every 13 minutes. The initial metabolic rate peak seen in all groups is a stress response caused by handling. Inset shows the delta energy expenditure, calculated as the mean post-CL316243 (minutes 26 to 312) minus the mean pre-CL316243 (minutes −78 to 0) energy expenditure in kcal/h. Data are mean ±SE, N = 8/group. Levels not connected by same letter are significantly different (P<0.05).

As a probe for cold-induced changes in adipose physiology, CL316243, a selective β3-adrenergic receptor agonist, was used to estimate the capacity for heat generation [19]. In the control mice, CL316243 increased TEE by ∼2.5-fold from the thermoneutral baseline (**Figure 2**). Cold-exposed mice had a greater increase in metabolic rate following injection of CL316243 than controls: 6.5% (not significant) and 19% (p<0.05) greater in the 1 and 4 hour groups, respectively (ICE#1) and 26% (p<0.05) and 29% (p<0.05) greater in the 4 and 8 hour groups, respectively (ICE#2, **Figure 2**).

To examine the transcriptional response to intermittent cold, markers of BAT activation were studied. Ucp1 mRNA was slightly (∼25%), but not significantly, increased in the 1 h, 4 h, and 8 h groups in ICE#1 and #2 (**Tables 1** **,** **2**). No significant difference in Ucp1 protein levels was detected (data not shown). Pgc1α, a transcriptional co-activator involved in mitochondrial biogenesis and in the induction of thermogenic genes [21], was significantly increased in both the 4 h groups (∼4-fold vs. control) and the 8 h group (∼7-fold vs. control) (**Tables 1** **,** **2**). No histologic differences were observed comparing iBAT from mice in the different treatment groups. These changes are consistent with a moderate increase in BAT activation with cold exposure.

We next investigated if the cold exposure induced beige/brite adipose tissue. As expected, at baseline BAT markers were very low in WAT (BAT: WAT ratios for Ucp1 of ∼4000 and Pgc1α of ∼10). Cold exposure had no consistent effect in the 1 h or 4 h groups, with a possible induction (not statistically significant) in the 8 h group (**Tables 1** **,** **2**). Likewise, two other beige/brite markers (Cd137 and Tmem26) showed no differences in gene expression (data not shown).

Tissue glucose uptake was measured using 2-deoxyglucose, which probes glucose transport predominantly in the 45 minutes after 2-deoxyglucose injection, weighted to the first minutes after injection due to exponential decay kinetics [22] (data not shown). In the 1 h cold treatment group with 2-deoxyglucose dosed at the start of the cold exposure, a 2-fold increase in gastrocnemius uptake was observed with no significant increase in BAT or other tissues (**Table 3**). These results suggest that muscle was a principal glucose-driven thermogenic tissue during this time interval, likely due to shivering. In the 4 h treatment group with 2-deoxyglucose dosed 3 hours after the start of cold exposure, 2-deoxyglucose uptake in BAT increased 39% (non-significant) and decreased in most other tissues (significantly so in quadriceps, iWAT, and spleen; **Table 3**). These results suggest that BAT thermogenesis was turned on in the 4 h group, with reduced glucose uptake in other tissues, consistent with a ‘steal’ phenomenon in which one tissue's uptake reduces the availability of glucose for uptake by other tissues.

**Table 3:** Tissue 2-deoxyglucose uptake.

	CON	1 h	4 h	
**Quadriceps (cpm/mg)**	8.6±2.1^A^	8.6±1.8^A^	4.0±0.5^B^	
**Gastrocnemius (cpm/mg)**	22.6±2.8^A^	46.2±10.7^B^	14.3±1.3^A^	
**Interscapular BAT (cpm/mg)**	37.9±6.0	30.4±6.5	52.5±10.8	
**Inguinal WAT (cpm/mg)**	1.2±0.1^A^	1.0±0.1^AB^	0.8±0.2^B^	
**Epididymal WAT (cpm/mg)**	3.4±0.6	4.6±1.5	2.6±0.4	
**Spleen (cpm/mg)**	21.1±1.7^A^	18.3±1.2^AB^	16.0±0.6^B^	

Mice in the ad lib fed state were administered [^14^C]2-deoxyglucose 1 hour prior to euthanasia. Specifically, this was at 22°C in the CON group, at the onset of 4°C in the 1 hour group, and 3 h into the 4°C treatment in the 4 hour group. Data are mean ±SE, N = 8/group. Levels not connected by same letter are significantly different (P<0.05).

Taken together, these data demonstrate that intermittent cold exposure increases thermogenic capacity, with modest increases in mRNA markers of BAT activation, and no consistent activation of beige/brite adipose tissue.

### Cold effect on energy homeostasis

**Figure 3:**
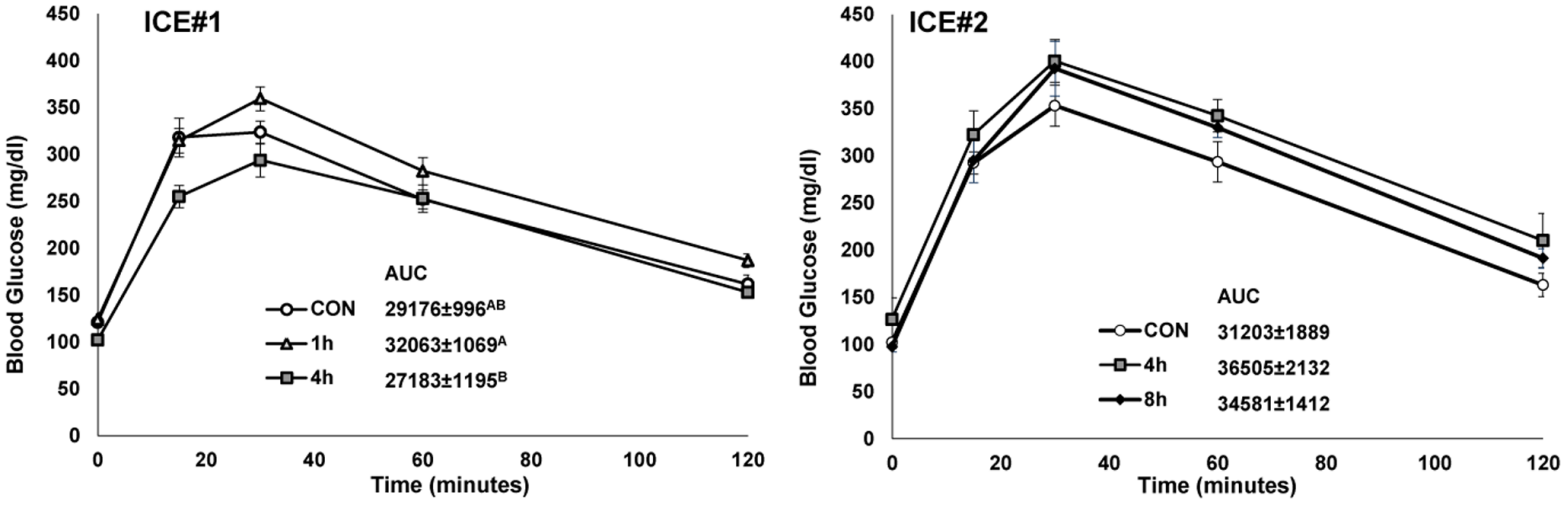
Transient improvement in glucose tolerance by cold exposure. In ICE#1 (top) and ICE#2 (bottom), intra-peritoneal glucose tolerance tests (1 g/kg) were performed in the overnight-fasted mice. In ICE#1, the ipGTT was conducted the day after cold exposure, while in ICE#2 it was conducted on the second day following cold exposure. The inset shows the mean area AUC in mg/dl•min ±SE, N = 8/group. Levels not connected by same letter are significantly different (P<0.05).

In ICE#1, an ipGTT was conducted the day following cold exposure to examine the effects of cold exposure on glucose tolerance. The 4 h group had decreased glucose concentrations at most time points and a significantly lower area under the curve (AUC) compared to the 1 h group (**Figure 3** **top**). In ICE#2, the ipGTT was performed two days after cold exposure to probe for longer lasting effects. With this paradigm, no significant effect was observed (**Figure 3** **bottom**), suggesting that there is a modest, transient beneficial effect of cold exposure on ipGTT.

In serum samples obtained at euthanasia after cold exposure, circulating free fatty acids were increased (**Table 1** **,** **2**), the result of lipolysis and fatty acid release from WAT. Insulin levels were slightly reduced by cold exposure, likely due to increased glucose utilization. No significant differences were observed in serum glucose, triglyceride, cholesterol, or adiponectin concentrations (**Table 1** **,** **2**). Regression analysis of leptin level vs. fat mass revealed the expected [23] positive correlation (data not shown). In addition, leptin concentrations were reduced by acute cold exposure indicating that cold, like fasting [24], reduces circulating leptin concentrations.

### No synergistic effects of cold exposure and AM251 on body weight, body composition, or food intake

**Figure 4:**
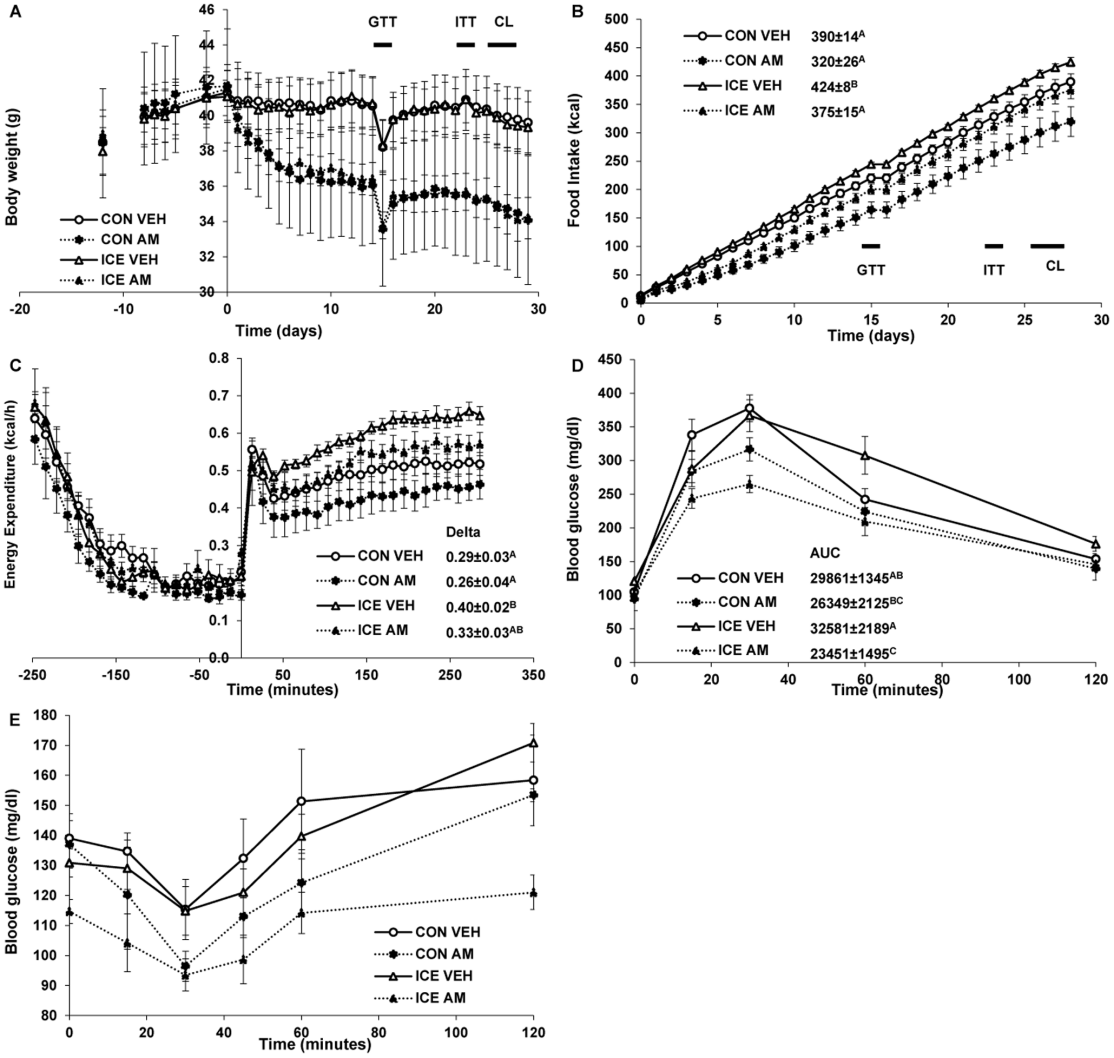
Effects of AM251 and cold exposure. Mice were administered vehicle or AM251 (3 mg/kg/day) by oral gavage. A: Caloric intake and B: body weight were measured three times per week on days of cold exposure. C: Mice at 30°C were administered CL316243 (100 µg/kg) at time 0 as described in Experimental Procedures (the prior AM251 was given at −24 hours). Inset shows the delta TEE, calculated as in Figure 2. 2-way ANOVA showed significant temperature (p<0.01) and drug (p<0.05) effects with no significant interaction (p = 0.62). D: Intra-peritoneal glucose tolerance tests (1 g/kg) were performed in overnight-fasted mice of the indicated treatment group two days after cold exposure. AUC numbers are in mg/dl•minute. E. Insulin tolerance test. Insulin (0.75 U/kg) was injected and blood glucose measured at the indicated times. GTT =  intraperitoneal glucose tolerance test. CL =  CL316243 experiment. ITT =  insulin tolerance test. AM251 was administered 24 h prior to the GTT and ITT. In D & E, a poor-responding outlier in the CON AM group was removed from the analysis. If included in the ipGTT AUC, this group's AUC is 29304 ±3382 mg/dl min and the CON AM significantly different from the ICE AM group. All data are mean ±SE. N = 6/group. Levels not connected by same letter are significantly different (P<0.05).

Since cold exposure increases food intake, we tried combining cold exposure with an obesity drug that reduces food intake. In ICE#3, we tested, individually and in combination, 4-hour cold exposure and AM251, a cannabinoid receptor 1 (CB1) inverse agonist that both reduces food intake and increases metabolic rate [25]. The AM251 dose (3 mg/kg/day) was chosen as efficacious, but well below maximum efficacy [26], thereby allowing room in the assay to detect additional weight loss. AM251 caused rapid and sustained weight loss that was similar in the cold exposed (ICE AM, −16±2%, p<0.05) and control (CON AM, −17±2%, p<0.05) groups (**Figure 4A** and **Table 4**). Total fat mass and inguinal and epididymal fat pad weights were reduced by ∼30% (P<0.05) in AM251 treated animals (**Table 4**). Decreased fat mass accounted for nearly all the weight loss in the drug treated animals.

**Table 4:** ICE#3, effect of daily AM251 and of 4 hours of cold exposure three times per week.

	CON VEH	CON AM	ICE VEH	ICE AM
**Body weight (g)**	39.6±1.8^B^	34.1±3.6^A^	39.3±1.4^B^	34.2±1.2^A^
**Fat mass (g)**	14.1±1.2^B^	9.7±2.6^A^	14.2±1.4^B^	9.8±1.0^A^
**Fat free mass (g)**	25.8±0.7	25±0.9	25.3±0.4	24.9±0.7
**Inguinal WAT mass (g)**	1.8±0.3^B^	1.19±0.43^A^	2.00±0.31^B^	1.26±0.16^A^
**Epididymal WAT mass (g)**	1.75±0.08^B^	1.26±0.24^A^	2.02±0.05^B^	1.29±0.10^A^
**Interscapular BAT mass (g)**	0.18±23	0.18±37	0.22±16	0.18±16
**Liver mass (g)**	1.4±0.1	1.4±0.2	1.4±0.1	1.2±0.1
**TEE (kcal/24 h)**	13.13±0.28^A^	13.07±0.30^A^	14.74±0.28^B^	14.94±0.28^B^
**BAT Pgc1α RNA**	1.00±0.21	1.19±0.21	0.90±0.16	0.99±0.13
**BAT Ucp1 RNA**	1.00±0.20	1.18±0.18	1.13±0.18	1.15±0.11
**WAT Pgc1α RNA**	1.00±0.11	0.96±0.16	1.49±0.22	1.16±0.07
**WAT Ucp1 RNA**	1.00±0.45	1.70±1.11	3.95±3.35	2.26±1.32
**Free fatty acids (mM)**	0.41±0.03 ^A^	0.40±0.03 ^A^	0.25±0.01 ^B^	0.26±0.03 ^B^
**Triglyceride (mg/dl)**	42.3±2.4 ^A^	47.7±2.5 ^A^	35.2±2.0 ^B^	32.2±1.5 B
**Serum Glucose (mg/dl)**	166±18	170±10	176±13	162±12
**Cholesterol (mg/dl)**	116±6	99±13	126±5	106±14
**Insulin (ng/ml)**	0.86±0.17	0.82±0.31	1.06±0.25	1.09±0.34
**Adiponectin (µg/ml)**	18.1±1.0	18.0±0.7	17.9±0.9	18.2±1.3
**Leptin (ng/ml)**	54±11	60±15	72±7	58±18
**T3 (ng/ml)**	2.03±0.05	2.13±0.18	2.09±0.12	2.04±0.10
**T4 (µg/dl)**	2.23±0.17 ^B^	2.52±0.21 ^A,B^	2.81±0.15 ^A^	2.76±0.21 ^A,B^
**β-hydroxybutyrate (mM)**	0.60±0.03 ^A,B^	0.71±0.05 ^A^	0.48±0.07 ^B^	0.50±0.03 ^B^
**D-Lactate (mM)**	0.65±0.02 ^A,B^	0.82±0.06 ^A^	0.51±0.09 ^B^	0.58±0.04 ^B^
**FGF-21 (pg/ml)**	167±43	289±90	186±47	187±86
**IGF-1 (ng/ml)**	271±21	259±16	291±18	258±9

Tissue weights, mRNA levels, and serum analytes were taken at the end of the experiment. TEE was calculated as described in Materials and Methods using the energy balance method over the full 28 days of the experiment. Gene expression values are reported relative to CON VEH. Data are mean ±SE, N = 6/group. Levels not connected by same letter are significantly different (P<0.05).

As expected, cumulative food intake was significantly decreased by AM251 in CON mice. AM251 also decreased food intake in cold exposed mice, but did not inhibit the compensatory increase in food intake caused by the elevated TEE of cold exposure (**Figure 4B**). 2-way ANOVA analysis of TEE revealed that cold exposure significantly increased TEE but AM251 did not (**Table 4**). Thus, there is no evidence for augmented (or reduced) benefits from combining cold exposure and AM251 on body weight, fat mass, or food intake.

### AM251 decreases CL316243-induced energy expenditure but does not affect BAT specific markers

AM251 is reported to cause weight loss partially through up-regulation of Ucp1 in BAT [27]. However, in AM251-treated mice, CL316243-induced energy expenditure was reduced compared to controls (**Figure 4C**). The decreased response to CL316243 is likely due to less lipolysis from the reduced fat mass of the AM251-treated animals. Gene expression of both *Pgc1α* and *Ucp1* in BAT showed no significant differences among the groups. Analysis by ANOVA revealed a significant effect of prior cold exposure to increase (p<0.05) and of AM251 treatment to decrease (p<0.05) CL316243-stimulated energy expenditure, but no interaction (p = 0.63) (**Figure 4C**).

### Combined effect of AM251 and cold on glucose and insulin tolerance

AM251 improves impaired glucose homeostasis [27], but how a combination of intermittent cold and AM251 administration affects such parameters is not known. ipGTT and ITT were performed two days after cold exposure, as in ICE#2 to probe for a durable effect. Glucose tolerance was significantly improved in the ICE AM and CON AM groups at the 30- and 60-minute time points compared to the ICE VEH group (**Figure 4D**). 2-way ANOVA using drug (±AM251) and cold exposure (±ICE) as independent variables and the AUC as the dependent variable, revealed a significant drug effect (p = 0.035) but no cold effect or interaction. Non-fasting serum glucose concentrations were significantly lower in the ICE AM compared to other groups at the beginning of the ITT (**Figure 4E**). Although CON AM serum glucose concentrations started significantly higher than ICE AM, they reached nearly identical concentrations 30 minutes following insulin injection (96.4±5.0 vs. 93.5±5.4 mg/dl respectively). Taken together, these data demonstrate that AM251 significantly decreases body weight and fat mass with beneficial effects on ipGTT and ITT but no clear synergistic effects when combined with cold exposure.

### Effect of AM251 and cold exposure on hormones and metabolites

In ICE#3, the mice were not exposed to cold at the terminal sampling, unlike ICE#1 and ICE#2, allowing differentiation between acute and more persistent effects of cold exposure. Circulating free fatty acid and triglyceride concentrations were significantly lower in ICE vs. CON mice irrespective of drug treatment (**Table 4**). β-hydroxybutyrate and D-lactate concentrations were lower in both ICE AM and ICE VEH when compared to CON AM but not CON VEH. Insulin was slightly but not significantly elevated in the two ICE groups compared to CON mice. No significant differences were observed in serum glucose, cholesterol, adiponectin, leptin, T3, FGF-21, or IGF-1 when compared with CON mice.

## Discussion

We have investigated the metabolic and physiologic consequences of moderate doses of intermittent cold exposure in DIO mice. Cold exposure increases food intake, energy expenditure, thermogenic capacity, and expression of BAT activation genes. Despite activation of BAT, there were no changes in body weight or composition, yet there were transient improvements in glucose homeostasis.

In our experiments, the acute cold-induced increase in energy expenditure is large (∼2 fold), but the overall increase in daily TEE is modest (4–12%). The appearance of brown adipocytes in typically white adipose depots occurs with prolonged exposure to cold temperatures [28], [29]. The current study produced only mild changes in interscapular BAT and no browning of the inguinal fat pad, results that are consistent with the modest level of additional cold challenge. Three questions are highlighted by our observations.

### How does intermittent BAT activation compare to exercise?

Initiating low levels of exercise in sedentary rats slightly reduced body weight and food intake. With higher exercise levels, weight remained stable while food intake rose, nearly doubling [30]. Like the sedentary state, mice housed at thermoneutrality are heavier than those housed at room temperature [31]–[33], supporting the parallel between muscle exercise and BAT activation. In our experiments, the control mice were housed at 22°C, below the thermoneutral zone, so the additional cold exposure is analogous to increasing exercise in mice that are already getting some exercise. It is plausible that intermittent cold exposure in mice otherwise housed at thermoneutrality would produce a body weight reduction.

In DIO mice, exercise protected against weight gain despite increased food intake, reduced adipose tissue inflammation, and improved insulin sensitivity [34]. Human epidemiologic evidence suggests that exercise training in obese patients improves glucose control and reduces inflammation, independent of weight loss [35]. It is unknown if cold-induced BAT activation would do the same, either by a non-specific increase in metabolic demand, or via specific BAT hormones, analogous to irisin from muscle [36] or leptin and adiponectin from adipose tissue. However, the observation of transiently improved glucose tolerance is an encouraging sign. The improvement in insulin sensitivity with exercise in the absence of weight loss occurs in muscle [37]; whether the improvement with cold exposure occurs directly in BAT is not known.

Cold exposure will always increase metabolism, whether via BAT activation or shivering [38]. BAT is efficient in generating heat via dissipating the proton motive force. Shivering involves the work of muscle contraction, so some of the energy is used for force production and this physical work/kinetic energy reduces the fraction of energy spent on heat production [39].

### Is BAT activation *per se* expected to reduce body weight?

Prior experiments suggest that increases in BAT quantity and/or activation can cause weight loss. Examples include BAT activation by transgenic manipulation [9], BAT transplantation [10], and β3-adrenergic agonist treatment [40]–[42]. The effect of BAT activation depends on whether the activation increases energy expenditure beyond the endogenous level, either by large increases in thermogenesis, or by affecting regulatory mechanisms. Long term increased energy expenditure is expected to be balanced by increased food intake to avoid eventual starvation. Indeed, in one study using CL316243, an initial reduction in food intake was followed by an increase over baseline [43]. Studies using chronic β3-adrenergic agonist treatment typically find little (or transient) weight loss and an increase in food intake [19], [41]–[43]. The protection of body weight is not limited to β3-adrenergic agonist stimulation or modest doses of cold—mice chronically exposed to −3°C maintain their body weight despite a 4-fold increase in metabolic rate [15]. In summary, achieving weight loss depends on how food intake compensates for the increase in energy expenditure, so BAT activation *per se* is not sufficient for weight loss.

### Are there strategies for using modest cold exposure that would ameliorate obesity and its metabolic consequences?

Successful therapeutic use of cold exposure requires blunting the accompanying increase in food intake. To address this issue, we investigated combining cold exposure with a weight loss drug, AM251, a cannabinoid receptor inverse agonist that reduces food intake. However, while both cold and AM251 maintained their individual effects, the combination did not yield augmented benefit. Another possible approach would be to supplement leptin levels. Cold stress induces central nervous system leptin receptor gene expression [44] and decreases WAT leptin [45]. Leptin replacement to the levels present in the absence of cold exposure would address this idea.

Is the mouse a relevant model for human energy homeostasis? Due to their large surface area-to-volume ratio, small mammals are exquisitely sensitive to environmental temperature and capable of drastically increasing BAT thermogenesis and food intake in response to decreases in ambient temperature. While it has been known since Lavoisier (ca 1790) that food intake and metabolic rate are increased by cold in humans [46], only relatively recently was it established that BAT is contributing to the cold-induced increase in thermogenesis in adult humans. Thus, while the magnitude of the effect is dramatically different, the biological insights from the mouse should provide fruitful investigative paths vis-à-vis BAT activation and function in humans. For example one of two recent human cold exposure trials did observe a reduction in fat mass [7], [8].

In summary, modest intermittent cold exposure does not reduce body weight or fat mass in mice but appears to transiently improve glucose homeostasis. The stimulation of BAT by cold has similarities to the stimulation of muscle by physical activity. Reducing the compensatory increase in food intake seen with cold exposure would be an effective way to achieve weight loss and improve metabolic status. Devising optimal use of cold exposure requires understanding how to combine it with exercise, food restriction, and pharmacologic therapy.

## References

[1] Gordon CJ (2012) Thermal physiology of laboratory mice: Defining thermoneutrality. J Thermal Biol 37: 654–685.

[2] van Marken Lichtenbelt WD, Vanhommerig JW, Smulders NM, Drossaerts JM, Kemerink GJ, et al (2009) Cold-activated brown adipose tissue in healthy men. N Engl J Med 360: 1500–1508. 19357405 10.1056/NEJMoa0808718

[3] Cypess AM, Lehman S, Williams G, Tal I, Rodman D, et al (2009) Identification and importance of brown adipose tissue in adult humans. N Engl J Med 360: 1509–1517. 19357406 10.1056/NEJMoa0810780 PMC2859951

[4] Virtanen KA, Lidell ME, Orava J, Heglind M, Westergren R, et al (2009) Functional brown adipose tissue in healthy adults. N Engl J Med 360: 1518–1525. 19357407 10.1056/NEJMoa0808949

[5] Kajimura S, Seale P, Spiegelman BM (2010) Transcriptional control of brown fat development. Cell Metab 11: 257–262. 20374957 10.1016/j.cmet.2010.03.005 PMC2857670

[6] Kozak LP (2010) Brown fat and the myth of diet-induced thermogenesis. Cell Metab 11: 263–267. 20374958 10.1016/j.cmet.2010.03.009 PMC2867325

[7] Yoneshiro T, Aita S, Matsushita M, Kayahara T, Kameya T, et al (2013) Recruited brown adipose tissue as an antiobesity agent in humans. J Clin Invest 123: 3404–3408. 23867622 10.1172/JCI67803 PMC3726164

[8] van der Lans AA, Hoeks J, Brans B, Vijgen GH, Visser MG, et al (2013) Cold acclimation recruits human brown fat and increases nonshivering thermogenesis. J Clin Invest 123: 3395–3403. 23867626 10.1172/JCI68993 PMC3726172

[9] Kopecky J, Clarke G, Enerback S, Spiegelman B, Kozak LP (1995) Expression of the mitochondrial uncoupling protein gene from the aP2 gene promoter prevents genetic obesity. J Clin Invest 96: 2914–2923. 8675663 10.1172/JCI118363 PMC186003

[10] Stanford KI, Middelbeek RJ, Townsend KL, An D, Nygaard EB, et al (2013) Brown adipose tissue regulates glucose homeostasis and insulin sensitivity. J Clin Invest 123: 215–223. 23221344 10.1172/JCI62308 PMC3533266

[11] Grujic D, Susulic VS, Harper ME, Himms-Hagen J, Cunningham BA, et al (1997) Beta3-adrenergic receptors on white and brown adipocytes mediate beta3-selective agonist-induced effects on energy expenditure, insulin secretion, and food intake. A study using transgenic and gene knockout mice. J Biol Chem 272: 17686–17693. 9211919 10.1074/jbc.272.28.17686

[12] Robidoux J, Martin TL, Collins S (2004) Beta-adrenergic receptors and regulation of energy expenditure: a family affair. Annu Rev Pharmacol Toxicol 44: 297–323. 14744248 10.1146/annurev.pharmtox.44.101802.121659

[13] Bing C, Frankish HM, Pickavance L, Wang Q, Hopkins DF, et al (1998) Hyperphagia in cold-exposed rats is accompanied by decreased plasma leptin but unchanged hypothalamic NPY. Am J Physiol 274: R62–68. 9458899 10.1152/ajpregu.1998.274.1.R62

[14] Holloszy JO, Smith EK (1986) Longevity of cold-exposed rats: a reevaluation of the “rate-of-living theory”. J Appl Physiol 61: 1656–1660. 3781978 10.1152/jappl.1986.61.5.1656

[15] Barnett SA (1965) Adaptation of Mice to Cold. Biol Rev Camb Philos Soc 40: 5–51. 14277936 10.1111/j.1469-185x.1965.tb00794.x

[16] Shefer VI, Talan MI (1997) Change in heat loss as a part of adaptation to repeated cold exposures in adult and aged male C57BL/6J mice. Exp Gerontol 32: 325–332. 9193900 10.1016/s0531-5565(96)00131-3

[17] Talan MI, Engel BT, Whitaker JR (1985) A longitudinal study of tolerance to cold stress among C57BL/6J mice. J Gerontol 40: 8–14. 3965565 10.1093/geronj/40.1.8

[18] Ravussin Y, Gutman R, Leduc CA, Leibel RL (2013) Estimating energy expenditure in mice using an energy balance technique. Int J Obes (Lond) 37: 473. 10.1038/ijo.2012.105 PMC3697837 22751256

[19] Cannon B, Nedergaard J (2004) Brown adipose tissue: function and physiological significance. Physiol Rev 84: 277–359. 14715917 10.1152/physrev.00015.2003

[20] Xiao C, Kim HS, Lahusen T, Wang RH, Xu X, et al (2010) SIRT6 deficiency results in severe hypoglycemia by enhancing both basal and insulin-stimulated glucose uptake in mice. J Biol Chem 285: 36776–36784. 20847051 10.1074/jbc.M110.168039 PMC2978606

[21] Uldry M, Yang W, St-Pierre J, Lin J, Seale P, et al (2006) Complementary action of the PGC-1 coactivators in mitochondrial biogenesis and brown fat differentiation. Cell Metab 3: 333–341. 16679291 10.1016/j.cmet.2006.04.002

[22] Conti PS, Sordillo PP, Sordillo EM, Schmall B (1986) Tumor localization of the metabolically trapped radiolabeled substrates 2-deoxy-D-glucose and aminocyclopentanecarboxylic acid in human melanoma heterotransplants. Am J Clin Oncol 9: 537–540. 3491534 10.1097/00000421-198612000-00014

[23] Rosenbaum M, Nicolson M, Hirsch J, Murphy E, Chu F, et al (1997) Effects of weight change on plasma leptin concentrations and energy expenditure. J Clin Endocrinol Metab 82: 3647–3654. 9360521 10.1210/jcem.82.11.4390

[24] Ahima RS, Prabakaran D, Mantzoros C, Qu D, Lowell B, et al (1996) Role of leptin in the neuroendocrine response to fasting. Nature 382: 250–252. 8717038 10.1038/382250a0

[25] Hildebrandt AL, Kelly-Sullivan DM, Black SC (2003) Antiobesity effects of chronic cannabinoid CB1 receptor antagonist treatment in diet-induced obese mice. Eur J Pharmacol 462: 125–132. 12591104 10.1016/s0014-2999(03)01343-8

[26] McLaughlin PJ, Winston KM, Limebeer CL, Parker LA, Makriyannis A, et al (2005) The cannabinoid CB1 antagonist AM 251 produces food avoidance and behaviors associated with nausea but does not impair feeding efficiency in rats. Psychopharmacology (Berl) 180: 286–293. 15948012 10.1007/s00213-005-2171-0

[27] Judge MK, Zhang Y, Scarpace PJ (2009) Responses to the cannabinoid receptor-1 antagonist, AM251, are more robust with age and with high-fat feeding. J Endocrinol 203: 281–290. 19679649 10.1677/JOE-09-0210

[28] Young P, Arch JR, Ashwell M (1984) Brown adipose tissue in the parametrial fat pad of the mouse. FEBS Lett 167: 10–14. 6698197 10.1016/0014-5793(84)80822-4

[29] Wu J, Cohen P, Spiegelman BM (2013) Adaptive thermogenesis in adipocytes: is beige the new brown? Genes Dev 27: 234–250. 23388824 10.1101/gad.211649.112 PMC3576510

[30] Mayer J, Marshall NB, Vitale JJ, Christensen JH, Mashayekhi MB, et al (1954) Exercise, food intake and body weight in normal rats and genetically obese adult mice. Am J Physiol 177: 544–548. 13158612 10.1152/ajplegacy.1954.177.3.544

[31] Castillo M, Hall JA, Correa-Medina M, Ueta C, Kang HW, et al (2011) Disruption of thyroid hormone activation in type 2 deiodinase knockout mice causes obesity with glucose intolerance and liver steatosis only at thermoneutrality. Diabetes 60: 1082–1089. 21335378 10.2337/db10-0758 PMC3064082

[32] Nikonova L, Koza RA, Mendoza T, Chao PM, Curley JP, et al (2008) Mesoderm-specific transcript is associated with fat mass expansion in response to a positive energy balance. FASEB J 22: 3925–3937. 18644838 10.1096/fj.08-108266 PMC2574032

[33] Rippe C, Berger K, Boiers C, Ricquier D, Erlanson-Albertsson C (2000) Effect of high-fat diet, surrounding temperature, and enterostatin on uncoupling protein gene expression. Am J Physiol Endocrinol Metab 279: E293–300. 10913028 10.1152/ajpendo.2000.279.2.E293

[34] Bradley RL, Jeon JY, Liu FF, Maratos-Flier E (2008) Voluntary exercise improves insulin sensitivity and adipose tissue inflammation in diet-induced obese mice. Am J Physiol Endocrinol Metab 295: E586–594. 18577694 10.1152/ajpendo.00309.2007 PMC2536732

[35] Hamer M, O'Donovan G (2010) Cardiorespiratory fitness and metabolic risk factors in obesity. Curr Opin Lipidol 21: 1–7. 19770655 10.1097/MOL.0b013e328331dd21

[36] Bostrom P, Wu J, Jedrychowski MP, Korde A, Ye L, et al (2012) A PGC1-alpha-dependent myokine that drives brown-fat-like development of white fat and thermogenesis. Nature 481: 463–468. 22237023 10.1038/nature10777 PMC3522098

[37] Goodyear LJ, Kahn BB (1998) Exercise, glucose transport, and insulin sensitivity. Annu Rev Med 49: 235–261. 9509261 10.1146/annurev.med.49.1.235

[38] Ukropec J, Anunciado RP, Ravussin Y, Hulver MW, Kozak LP (2006) UCP1-independent thermogenesis in white adipose tissue of cold-acclimated Ucp1-/- mice. J Biol Chem 281: 31894–31908. 16914547 10.1074/jbc.M606114200

[39] Haman F, Blondin DP, Imbeault MA, Maneshi A Metabolic requirements of shivering humans. Front Biosci (Schol Ed) 2: 1155–1168. 10.2741/s124 20515847

[40] Himms-Hagen J, Cui J, Danforth E Jr, Taatjes DJ, Lang SS, et al (1994) Effect of CL-316,243, a thermogenic beta 3-agonist, on energy balance and brown and white adipose tissues in rats. Am J Physiol 266: R1371–1382. 7910436 10.1152/ajpregu.1994.266.4.R1371

[41] Gavrilova O, Marcus-Samuels B, Reitman ML (2000) Lack of responses to a beta3-adrenergic agonist in lipoatrophic A-ZIP/F-1 mice. Diabetes 49: 1910–1916. 11078459 10.2337/diabetes.49.11.1910

[42] Yoshida T, Sakane N, Wakabayashi Y, Umekawa T, Kondo M (1994) Anti-obesity effect of CL 316,243, a highly specific beta 3-adrenoceptor agonist, in mice with monosodium-L-glutamate-induced obesity. Eur J Endocrinol 131: 97–102. 7913651 10.1530/eje.0.1310097

[43] Himms-Hagen J, Cui J, Danforth E Jr, Taatjes DJ, Lang SS, et al (1994) Effect of CL-316,243, a thermogenic beta 3-agonist, on energy balance and brown and white adipose tissues in rats. Am J Physiol 266: R1371–R1382. 7910436 10.1152/ajpregu.1994.266.4.R1371

[44] Mercer JG, Moar KM, Rayner DV, Trayhurn P, Hoggard N (1997) Regulation of leptin receptor and NPY gene expression in hypothalamus of leptin-treated obese (ob/ob) and cold-exposed lean mice. FEBS Lett 402: 185–188. 9037192 10.1016/s0014-5793(96)01525-6

[45] Trayhurn P, Duncan JS, Rayner DV (1995) Acute cold-induced suppression of ob (obese) gene expression in white adipose tissue of mice: mediation by the sympathetic system. Biochem J 311 (Pt 3): 729–733. 10.1042/bj3110729 PMC1136063 7487925

[46] Lusk G (1928) The science of nutrition. Philadelphia: W. B. Saunders Company. 844 p.

